# Mechanical, workability, economic and environmental properties of concrete with limestone calcined clay cement and recycled aggregates

**DOI:** 10.1038/s41598-025-97539-6

**Published:** 2025-04-23

**Authors:** Yong Yu, Mingjian Yang, Cheng Wan, Lang Lin

**Affiliations:** 1 School of Intelligent Transportation and Engineering, Guangzhou Maritime University, Guangzhou, 510725 China; 2 School of Civil Engineering and Transportation, South China University of Technology, Guangzhou, 510640 China; 3 School of Architecture and Engineering, Jiangxi Polytechnic University, Jiujiang, 332007 China; 4 School of Transportation, Civil Engineering and Architecture, Foshan University, Foshan, 528051 China

**Keywords:** Coarse and fine recycled aggregates, Limestone calcined clay cement, Mechanical strengths and elastic modulus, Unit cost and carbon footprint, Civil engineering, Mechanical engineering

## Abstract

Limestone calcined clay cement (LC3) and recycled aggregates (RAs) are important innovations in civil engineering, offering solutions to the industry’s energy and carbon challenges. However, limited research on their combined use has hindered widespread application. This study systematically evaluates LC3-based concrete with both coarse and fine RAs, using material testing, cost analysis and carbon footprint assessments to examine the effects of water-to-binder ratio, metakaolin-limestone powder synergy, RA content and particle quality on workability, compressive strength, elastic modulus, splitting-tensile strength, unit cost and environmental impact, while establishing clear quantitative relationships. Key findings include: (i) Using up to 50% metakaolin-limestone powder by mass, 30% coarse RA and 20% recycled sand by volume results in minimal reductions in 28-day strength and elastic modulus. However, exceeding 70%, 50% and 40%, respectively, leads to significant mechanical deterioration. (ii) LC3 concrete has 4-15% lower strength than Portland cement concrete at 14 days due to slower pozzolanic reactions, but strength levels equalize by 28 days. Similarly, recycled aggregate concrete shows lower early-age strength, with extended curing further highlighting performance differences, likely due to the residual mortar on recycled particles. (iii) Lifecycle analysis confirms that replacing 50% of the binder with metakaolin-limestone powder reduces carbon emissions by 29.9% and costs by 12.4% for strength-equivalent concrete. In contrast, RAs offer only modest sustainability benefits, with 20% recycled sand and 30% coarse RA increasing emissions by 5.4% and 2.8%, respectively, while keeping costs stable. (iv) The quality of RA particles plays a key role in their sustainable application. Additionally, transportation distance significantly affects the circular benefits of RA use but has a limited impact on the sustainability of metakaolin-limestone powder.

## Introduction

Over the past 30 years, civil engineering has advanced rapidly. Developing nations like India and China have fueled large-scale urban projects, while aging infrastructure in developed countries has required urgent upgrades. This growth has made construction a major consumer of resources and energy, as well as a key source of emissions and waste. By 2021, the industry had used 14 billion cubic meters of concrete annually, emitted 10 billion tons of CO₂-equivalent gases, and generated 7 billion tons of construction waste^1–3^.

Cement, a key construction material, has a heavy environmental impact. Its production emits 2.3 billion tons of greenhouse gases each year^4^, mainly from limestone calcination at high temperatures. This process releases large amounts of CO₂ and SO₂ while consuming significant fossil fuels like coal and natural gas, with each ton of cement producing 0.8–1 ton of CO₂-equivalent emissions^5^.

To curb environmental pollution, supplementary cementitious materials (SCMs) have gained attention for reducing cement use while enhancing concrete performance. Fly ash, a byproduct of coal combustion, improves impermeability and chemical resistance; slag, from blast furnaces, boosts long-term strength and durability, especially in high-strength concrete; and silica fume, a ferrosilicon byproduct, increases density, crack resistance and compressive strength^6^. However, limited availability and rising costs hinder their large-scale replacement of cement worldwide.

To tackle this challenge, developing low-carbon binders has become crucial, with limestone calcined clay cement (LC3) emerging as a promising solution. Developed by Professor Karen Scrivener’s team^7–12^, LC3 has gained global attention for its unique advantages. Its typical composition includes 50% clinker, 30% clay (e.g., metakaolin), 15% limestone powder and 5% gypsum^13^. By replacing a significant portion of clinker with abundant, low-energy materials like clay and limestone powder, LC3 reduces calcination temperatures, lowers energy consumption, and cuts carbon emissions. During hydration, these components react with clinker to form additional cementitious compounds, enhancing density and performance^14^. As a result, LC3 offers higher strength, improved durability and greater resistance to permeability and chemical attack, making it ideal for humid and corrosive environments^15,16^.

Aggregates make up 65–80% of concrete by volume, and relying solely on natural sand and gravel could deplete mineral resources as demand grows. To address this, recycled aggregates (RAs) have gained traction. Sourced from demolished concrete, they are mechanically crushed, stripped of steel reinforcements, and reused in new concrete production^17^. This process reduces the need for blasting, excavation and long-distance transport, cutting energy use and carbon emissions. It also helps manage construction waste, easing landfill pressure and benefiting other industries^18^.

After nearly two decades of research, coarse RAs have been successfully used in load-bearing structures, while recycled sand is undergoing small-scale trials. China’s RAC technical code^19^ permits up to 50% coarse RAs and 30% recycled sand by volume in load-bearing components. Current studies focus on improving RAC through techniques like two-stage mixing^20^, the equivalent volume mortar method^21^, fiber-nanoparticle modification^22^ and compaction pouring^23^ to address RAs’ inherent flaws—such as the surface cement paste layer (mortar for coarse RAs, slurry for fine RAs) and internal cracks. These defects lower RA quality, negatively impacting concrete’s mechanical properties and durability^24^.

Building on this foundation, the combined use of LC3 and RAs offers a sustainable, low-carbon solution to resource depletion and environmental challenges in construction. In recent years, researchers have increasingly explored the material and structural properties of this innovative mix. For example, Ding et al.^25^ studied LC3 and recycled sand in engineered cementitious composites (ECC) to reduce costs, examining how the fly ash-to-LC3 ratio and recycled sand fineness affect compressive strength, tensile strength and ductility. Their findings showed that LC3 stabilized crack propagation, while recycled sand enhanced tensile strength by about 8%. Zhou’s team^26^ investigated the effects of combining seawater, sea sand, coarse RAs and LC3 on concrete strength, as well as their impact on carbon emissions and energy use at both material and structural levels. More recently, Alghamdi et al.^27,28^ studied the impact of LC3 and fine RAs on mortar strength, using electron microscopy and X-ray diffraction to analyze the microstructural changes in the concrete. These studies have deepened understanding of LC3-based RAC, reinforcing its practical potential.

However, to further advance its application, additional research is necessary to explore the performance characteristics of LC3-based RAC, as several key issues in the engineering field remain insufficiently addressed^29,30^. For instance, the variability in RA quality significantly impacts both the mechanical properties and long-term sustainability of concrete. Furthermore, the distinct composition of LC3 and RA affects concrete performance in different ways over time. Additionally, given the concentration of production facilities in specific regions, it is vital to evaluate whether these raw materials consistently offer economic and environmental benefits across different project locations. These phenomena have yet to be quantitatively assessed.

In this context, the present study evaluates the workability, mechanical properties, economic feasibility and environmental impact of LC3-based RAC. It consists of two main phases: experimentation and analysis. In the experimental phase, key variables—such as the water-to-binder ratio, metakaolin and limestone powder content, RA proportion, and the strength grade of the parent concrete—were analyzed for their effects on slump, compressive strength, elastic modulus and splitting tensile strength. Quantitative correlations were established between macro-scale material properties and internal composition. The analysis phase uses lifecycle assessment to compare the production costs and carbon emissions of LC3-based RAC with conventional ordinary Portland cement (OPC)-based concrete made with natural aggregates, emphasizing the benefits of metakaolin, limestone powder, and coarse and fine RAs. Meanwhile, the environmental impact of transportation distances for recycled and alternative materials will also be discussed.

Through these efforts, this study provides new insights in the following areas:

- The time-dependent development of strength and elastic modulus in LC3-based RAC is clearly presented, demonstrating significantly different early-stage mechanical characteristics from conventional concrete.
- Prediction formulas for the strength and deformation indices of LC3-based RAC are established.
- The sustainability of LC3-based RAC is explored in depth, with a detailed examination of how internal mix variables and external factors influence the economic and environmental indicators of recycled and alternative materials.

It is anticipated that the findings of this study will provide important support for the practical application of this novel concrete in engineering projects.

## Experimental program

### Raw material preparation

The raw materials used to prepare all cylindrical and cubic specimens included OPC, metakaolin, limestone powder, river sand, recycled sand, natural gravel, coarse RAs, and a superplasticizer.

**Fig. 1:**
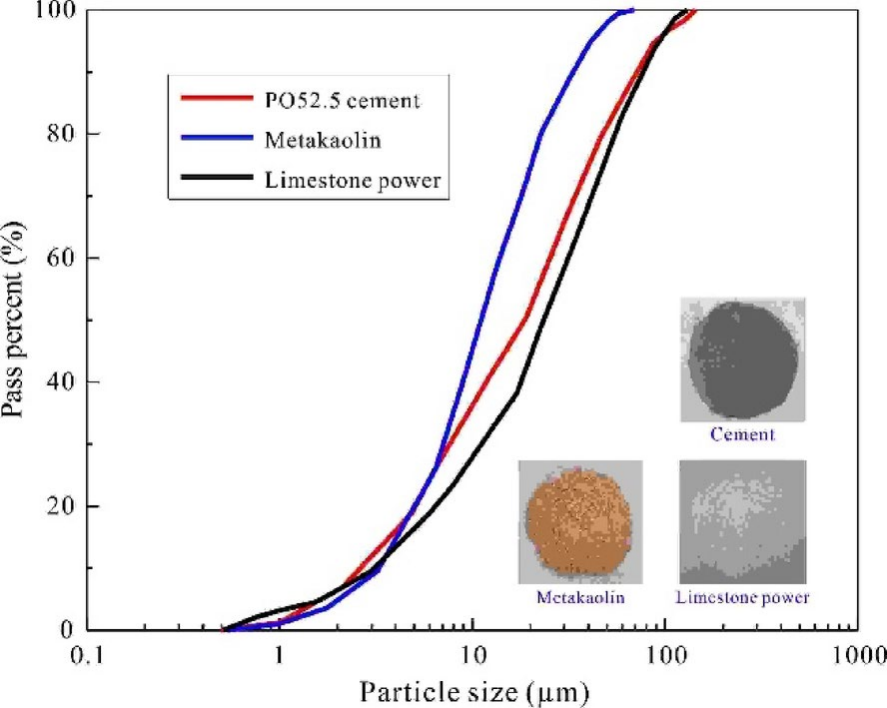
Grading curves of binders.

The OPC, which was sourced from Dongguan China Resources Cement Co., Ltd., had a grade of PO52.5 and consisted of 95% clinker and 5% gypsum. The 28-day compressive strength of the cement mortar was approximately 58.3 MPa, with a standard consistency of 29.8%. Metakaolin, obtained from Guangdong Yuanlei Powder Co., Ltd., was produced by calcining kaolin at around 750 °C to activate its pozzolanic properties. Limestone powder, purchased from Guangdong Ruihua Group Co., Ltd., contained nearly 98% calcium oxide. Table 1 presents the chemical composition of the three binders, which was determined via X-ray diffraction analysis, while Fig. 1 shows their morphology and particle gradation, measured using a dry sieving test in accordance with the GB/T 1345–2005 specification^31^.

**Fig. 2:**
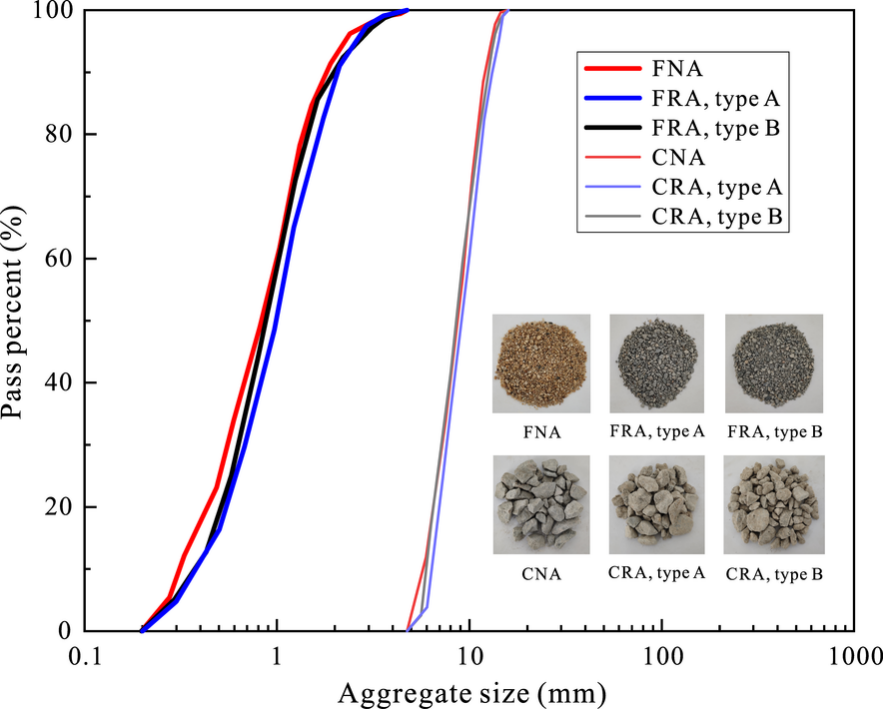
Grading curves of aggregate.

**Table 1:** Chemical constituents of binder materials.

Binder type	Composition (%)							
	SiO_2_	CaO	Al_2_O_3_	Fe_2_O_3_	MgO	K_2_O	Na_2_O	Others
PO52.5 cement	16.8	70.4	3.2	4.1	1.4	0.1	0.2	3.8
Metakaolin	52.1	0.2	38.2	1.4	0.4	3.6	0	4.1
Limestone power	0.3	97.6	0.1	0	0.6	0.1	0	1.3

River sand, which was sourced from a mining site in Dongjiang, Dongguan, had a density of 2530 kg/m^3^, a fineness modulus of 2.67 and an absorption rate of 1.32%, as determined by the oven-drying method in accordance with reference^32^. The natural gravel, a granite from Dalingshan Quarry in Dongguan, had a particle density of 2690 kg/m^3^, an absorption rate of 0.56% (measured by the oven-drying method per reference^32^) and a crushing index of 8.7% (determined using the load-bearing crush method per code^33^). Figure 2 shows the size distribution of the two types of natural aggregates.

This study used two types of fine and coarse RAs, both sourced from demolished concrete of different strength grades. Recycled sand A and coarse RA A were obtained through two-stage crushing of aged concrete slabs, with a parent concrete strength of approximately 21.5 MPa. Recycled sand B and coarse RA B originated from crushed concrete columns with a strength of 45.6 MPa. After crushing, the aggregates were sieved, washed, dried and mixed to match the natural aggregate gradation (Fig. 2). The natural densities of recycled sands A and B were approximately 2304 and 2390 kg/m^3^, with absorption rates of 13.15% and 8.96%, respectively. Coarse RAs A and B had densities of about 2530 and 2602 kg/m^3^, with absorption rates of 6.21% and 2.65%. The old mortar content was 56.2% and 34.5%, with crushing indices of 19.6% and 15.7%. Compared to natural aggregates, RAs exhibited significantly lower physical and mechanical properties.

The admixture was a light yellow, polycarboxylate-based superplasticizer from BASF, Germany. Based on a previous mix design study^34^, the optimal dosage ranged from 0.2 to 0.4% of the total binder content. Given the high water demand of LC3 and RAs, this study adopted the upper limit to maintain workability. Excessive water reducer could cause segregation, while insufficient dosage affected workability. Using the upper limit kept the slump of M15 and M17 concretes within 120–180 mm, ensuring good workability without further adjustment.

### Specimen design and manufacture

To investigate the material properties of LC3-based RAC, 18 mix proportions were designed and cast to comprehensively explore the effects of factors like the combined usage of metakaolin and limestone power, the volumetric replacement rates of fine and coarse RAs, strength grade of RAs and water-to-binder ratio. Table 2 presents the mix details for all concrete batches. It is important to note that the water listed in the table referred to effective water. For the dried RAs used in this study, additional water was typically added during concrete mix preparation. Following the method in^35^, the mass of this extra water was set at 70% of the saturated absorption of the coarse and fine RAs.

It is evident that all concrete mixtures in Table 2 were labeled with the notation “*x*-*y*-*zm*-*ln*”, where *x* represents the water-to-binder ratio, i.e., the ratio of effective water to the total mass of the three binders; *y* denotes the mass ratio of metakaolin and limestone powder replacing OPC, with the metakaolin-to-limestone powder ratio fixed at 2:1; *z* and *m* correspond to the volumetric replacement rate and type of fine RA; *l* and *n* represent the volumetric content and type of coarse RA.

A detailed comparison revealed the following: (i) M3, M4, M5 and M6 were primarily used to examine the effect of increasing the combined replacement ratio of metakaolin and limestone powder from 0 to 70%; (ii) M7, M5, M8 and M9 explored the effect of increasing the volumetric proportion of fine RA from 0 to 60%; (iii) M10, M5, M11 and M12 investigated the effect of increasing the volumetric proportion of coarse RA from 0 to 70%; (iv) M5, M13 and M14 examined the effect of the strength grade of RAs; (v) M15, M1 and M17, as well as M16, M5 and M18, studied the effect of varying the water-to-binder ratio from 0.32 to 0.55.

**Table 2:** Mix proportions for various kinds of LC3-based-RACs (kg/m^3^).

Mix no.	Mix notation	W	C	M	LP	FNA	FRA	CNA	CRA	SP
M1	0.45-0-0-0	242	535	0	0	814	0	808	0	2.1
M2	0.45-0.5-0-0	242	268	178	89	814	0	808	0	2.1
M3	0.45-0-0.2B-0.3B	242	535	0	0	651	154	566	234	2.1
M4	0.45-0.3-0.2B-0.3B	242	375	107	54	651	154	566	234	2.1
M5	0.45-0.5-0.2B-0.3B	242	268	178	89	651	154	566	234	2.1
M6	0.45-0.7-0.2B-0.3B	242	161	250	125	651	154	566	234	2.1
M7	0.45-0.5-0-0.3B	242	268	178	89	814	0	566	234	2.1
M8	0.45-0.5-0.4B-0.3B	242	268	178	89	488	308	566	234	2.1
M9	0.45-0.5-0.6B-0.3B	242	268	178	89	326	461	566	234	2.1
M10	0.45-0.5-0.2B-0	242	268	178	89	651	154	808	0	2.1
M11	0.45-0.5-0.2B-0.5B	242	268	178	89	651	154	404	391	2.1
M12	0.45-0.5-0.2B-0.7B	242	268	178	89	651	154	242	547	2.1
M13	0.45-0.5-0.2 A-0.3B	242	268	178	89	651	148	566	234	2.1
M14	0.45-0.5-0.2B-0.3 A	242	268	178	89	651	154	566	228	2.1
M15	0.32-0-0-0	192	600	0	0	814	0	808	0	2.4
M16	0.32-0.5-0.2B-0.3B	192	300	200	100	651	154	566	234	2.4
M17	0.55-0-0-0	281	511	0	0	814	0	808	0	2.0
M18	0.55-0.5-0.2B-0.3B	281	256	170	85	651	154	566	234	2.0

*W* water, *C* cement, *M* metakaolin, *LP* limestone power, *FNA* fine NA, *FRA* fine RA, *CNA* coarse NA, *CRA* coarse RA, *SP* superplasticizer.

In this study, a two-stage mixing method, based on literature^35^, was employed for all mixes. Initially, the aggregates were dry-mixed for 1 min, followed by the addition of half the water and all binders, mixing for 2 min. The remaining LC3 and superplasticizer were then added and mixed for another 2 min. This method, which ensured the binder slurry wraps around the RAs, significantly enhanced the concrete’s hardened properties^35^. After casting, the specimens were cured in a standard environment (20 ± 2 °C, 95% humidity) and subjected to mechanical testing once the designated curing period was reached.

For M1, M2, M7 and M10 mixes, compressive strength, elastic modulus and splitting tensile strength of cylindrical and cubic specimens were measured at 3, 7, 14, 28, 60, 90, 180 and 360 days. For the other mixes, these properties were measured only at 28 days. Compressive strength and elastic modulus were determined according to ASTM C39^36^, using 100 mm diameter, 200 mm height cylinders. Deformation at 0.5 times the cylinder height was measured with a high-precision displacement sensor (5 mm range, 0.001 mm resolution) at a fixed loading rate of 0.12 mm/min. Splitting-tensile strength was measured in accordance with GB/T 50,164^37^, using 100 mm cubes at a loading rate of 0.08 MPa/s.

## Experimental results and discussion

### Workability of fresh concrete

**Fig. 3:**
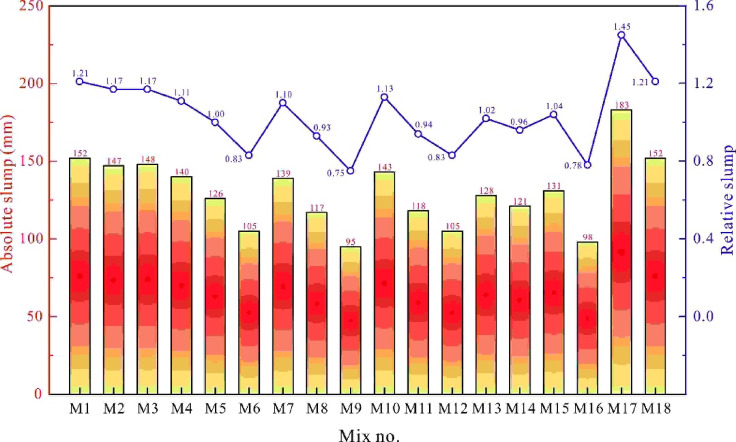
Slump measurements for different fresh LC3-based-RAC mixtures.

Previous studies^30,38,39^ have reported that both LC3 binder and RA significantly degrade the workability of concrete. Therefore, this study briefly explores the change in slump of fresh concrete when these two materials are used together and attempts to explain the underlying reasons. Figure 3 illustrates the slump values of 18 distinct concrete types. For comparative analysis, the figure also presents the relative slump values of various concretes benchmarked against mix M5.

As clearly demonstrated, the water-to-binder ratio was the primary determinant of concrete fluidity. As the ratio decreased from 0.55 to 0.32, the slump of OPC-based natural aggregate concrete decreased from 183 mm (M17) to 131 mm (M15), while the slump of LC3-based RAC decreased from 152 mm (M18) to 98 mm (M16). Comparatively, the latter exhibited a more pronounced decrease in workability (28.4% vs. 35.5%).

With a fixed water-to-binder ratio of 0.45, 20% recycled sand content and a 30% coarse RA replacement rate, increasing the combined substitution of metakaolin and limestone powder for cement from 0 to 70% resulted in a monotonous slump decline from 148 mm to 105 mm—a significant reduction of 29.1%. The decline accelerated notably beyond a 50% substitution rate, underscoring the need for careful application and precise design of LC3 to prevent unintended drawbacks despite its economic and environmental benefits. Guo et al.^26^ attributed the reduced fluidity of LC3-based concrete to two factors: the finer particle size of metakaolin relative to cement clinker, which required more water for pozzolanic reactions, and its high Al₂O₃ content, which rapidly generated abundant gel-like ettringite, significantly increasing paste viscosity.

RAs markedly impaired the workability of fresh concrete. For instance, increasing recycled sand content from 0 to 60% reduced the slump from 139 mm to 95 mm (a 31.7% decrease), while raising the coarse RA replacement from 0 to 70% reduced the slump from 143 mm to 105 mm (a 26.7% decrease). This degradation, particularly pronounced with recycled sand, likely resulted from aggregate geometry, as additional water had been preemptively added to counteract absorption effects. Yu et al.^39^ attributed this to the rougher surface of RAs, caused by adhered mortar, which increased particle movement resistance in the mortar matrix.

The slump of fresh concrete in mixes M5, M13 and M14 differed by no more than 5.4% (126 mm, 128 mm and 121 mm, respectively), demonstrating that RA particle quality had minimal impact on fresh-state workability. Except for M9 and M16, all mixes met the Chinese standard, which required a slump above 100 mm for flowing concrete. However, mixtures with higher contents of metakaolin, limestone powder or RA often exhibited reduced workability, requiring higher dosages or higher-quality water-reducing agents to meet construction requirements.

### Mechanical properties of Harden concrete

#### 28-day mechanical indicators

**Fig. 4:**
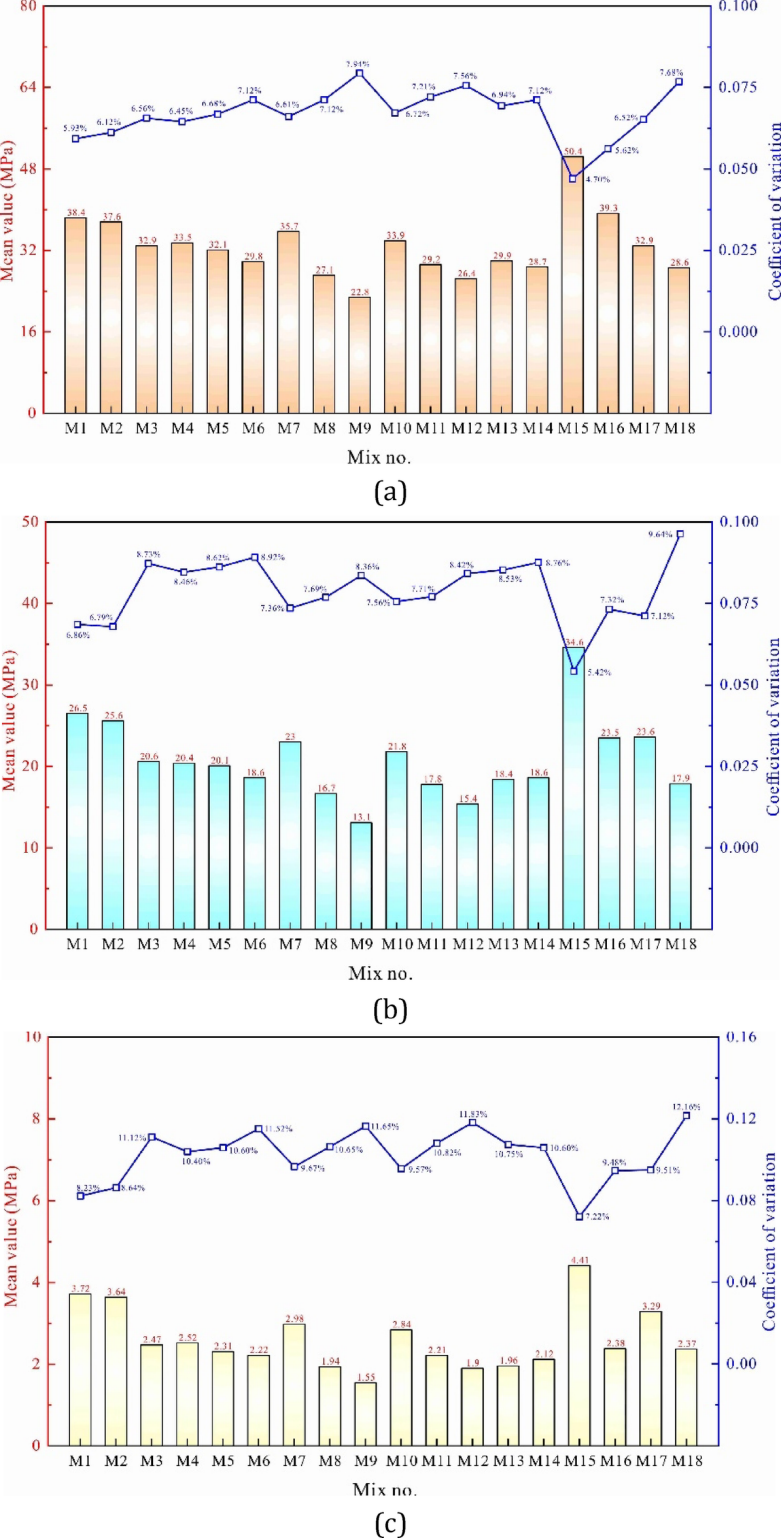
A 28-day mechanical indices for different LC3-based-RAC mixtures: (**a**) Compressive strength, (**b**) Elastic modulus, (**c**) Splitting-tensile strength.

Figure 4a illustrates the mean compressive strength and coefficient of variation for 18 types of concrete after a 28-day curing period, with each physical parameter representing the statistical characteristics derived from three parallel cylindrical specimens.

The results showed that the dosage of binder material had a significant impact on compressive strength. As the water-to-binder ratio decreased from 0.55 to 0.32, the compressive strength of OPC-based natural aggregate concrete increased from 32.9 to 50.4 MPa, marking a substantial rise of 53.2%. Similarly, the compressive strength of LC3-based RAC increased from 28.6 to 39.3 MPa, reflecting a 37.4% improvement. However, this concrete type exhibited a mitigated response to variations in the water-to-binder ratio. This phenomenon can be attributed to the substantial amount of adhered old mortar on RAs, which undergoes degradation under the denser encapsulation of new mortar, significantly constraining the performance enhancement typically facilitated by a reduced water-to-binder ratio.

The mean compressive strength of OPC-based RAC M3 was 32.9 MPa. When metakaolin and limestone powder were jointly used as partial cement replacements at substitution rates of 30% and 50%, the compressive strengths of LC3-based RACs M4 and M5 were 33.5 and 32.1 MPa, respectively, exhibiting marginal variations of 1.8% and − 2.4%. However, at a substitution rate of 70%, the mean compressive strength of M6 declined sharply to 29.8 MPa. These findings reveal that excessive replacement of cement with metakaolin and limestone powder undermines strength retention. This reduction likely results from the lower pozzolanic reactivity of these supplementary cementitious materials compared to clinker, which requires extended reaction times for complete hydration. Excessive incorporation exacerbates this limitation, further impairing concrete performance.

The incorporation of recycled sand significantly reduced compressive strength, with the decline becoming more pronounced as replacement levels increased. At recycled sand contents of 0, 20%, 40% and 60%, the 28-day compressive strengths of M7, M5, M8 and M9 were 35.7, 32.1, 27.1 and 22.8 MPa, respectively, corresponding to reductions of 10.1%, 24.1% and 36.1% at replacement rates of 20%, 40% and 60%. The increasing rate of decline suggests that the weaker adhered cement paste present on recycled sand, which replaces fine aggregate in bearing external loads, is inherently more prone to failure.

A similar trend was observed with coarse RA substitution, though its impact on strength was relatively less severe. At volumetric replacement rates of 30%, 50% and 70%, the 28-day compressive strengths of concretes M5, M11 and M12 decreased by 5.3%, 13.9% and 22.1%, respectively, compared to M10, with the rate of reduction progressively intensifying. This degradation is primarily attributed to the compromised quality of coarse RA, which contains adhered mortar, pre-existing fracture fissures, and embedded impurities, all of which degrade overall mechanical performance.

The strength grade of RA further influenced compressive strength, as evidenced by M5, M13 and M14, which had compressive strengths of 32.1, 29.9 and 28.7 MPa, respectively. The latter two exhibited reductions of 6.9% and 10.6% relative to M5, highlighting the detrimental effect of lower aggregate strength. Inferior aggregate quality exacerbates mechanical deficiencies, further compromising the performance of the resultant concrete.

Figure 4a also shows how different variables affect the variation in compressive strength. As the water-to-binder ratio, RA content or combined use of metakaolin and limestone powder increased, the compressive strength variation generally increased. Among the 18 concrete mixes in Table 1, M15 had the lowest compressive strength variation at 4.70%, while M9 had the highest at 7.94%, a difference of 3.24%. This highlights that using recycled or substitute materials may lead to instability in concrete performance, a point that requires attention from experts.

Figure 4b presents the mean elastic modulus and coefficient of variation for each concrete type. Compared to Fig. 4a, the coefficient of variation for elastic modulus was generally 0.4–2.1% higher than for compressive strength, likely due to the inherent variability in both natural and recycled aggregates. Key observations were as follows: (i) As the water-to-cement ratio decreased from 0.55 to 0.32, the elastic modulus of OPC-based natural aggregate concrete rose from 23.6 to 34.6 GPa, an increase of 46.6%. For LC3-based RAC, the elastic modulus increased from 17.9 to 23.5 GPa, a 31.8% rise. The former concrete type showed a more significant change in deformation due to variations in cementitious content, consistent with the trends observed in compressive strength. Furthermore, the effect of the water-to-binder ratio on the elastic modulus was less pronounced. (ii) As the combined replacement rate of metakaolin and limestone powder increased from 0 to 70%, the elastic modulus of LC3-based RAC decreased by only 9.7%, indicating that this factor had minimal impact on overall material deformation and could be disregarded in practical applications. (iii) The inclusion of RAs notably impacted the deformation modulus. For instance, when the volume replacement rate of fine RAs rose from 0 to 60%, the elastic modulus decreased from 23.0 to 13.1 GPa, a reduction of 43.0%. The rate of decrease was not linear—when the replacement rate increased from 0 to 20%, the modulus decreased by 12.6%, but as it rose from 20 to 40% and from 40 to 60%, the reductions accelerated to 14.8% and 15.7%, respectively. A similar trend was observed with coarse RAs, though their effect was somewhat less pronounced. (iv) The elastic moduli of hardened concrete mixes M5, M13 and M14 were 20.1, 18.4 and 18.6 GPa, respectively, showing a deviation of about 8.5%. This indicates that the quality of RA particles influences the deformation capacity of RAC, likely due to differences in the adhesive mortar ratio of various RAs.

Figure 4c presents the statistical characteristics of the splitting tensile strength for the 18 concrete types. Key observations were as follows: (i) The coefficient of variation for splitting tensile strength was notably higher than for compressive strength and elastic modulus, likely due to the impact of randomly distributed microcracks and pores on the tensile crack path^29^. (ii) Similar to elastic modulus, splitting tensile strength increased with a decrease in water-to-binder ratio, though the increase was smaller than in compressive strength. Additionally, LC3-based RAC exhibited less variation in splitting tensile strength for the same change in water-to-binder ratio. (iii) Replacing cement with metakaolin and limestone powder reduced splitting tensile strength, with the effect becoming more pronounced at substitution rates above 30%. At 50% and 70% substitution, the 28-day tensile strength decreased by 6.4% and 10.1%, respectively. (iv) The content and quality of RAs significantly influenced splitting tensile strength. For instance, as the volume replacement rate of fine RAs increased from 0 to 60%, the tensile strength dropped by 47.9%. When the compressive strength of the parent concrete decreased from 45.6 to 21.5 MPa, splitting tensile strength decreased by 15.2%. (v) An increase in water-to-binder ratio, higher use of metakaolin and limestone powder, and the addition of RAs all increased the variability of the splitting tensile strength.

#### Temporal development of mechanical properties

**Fig. 5:**
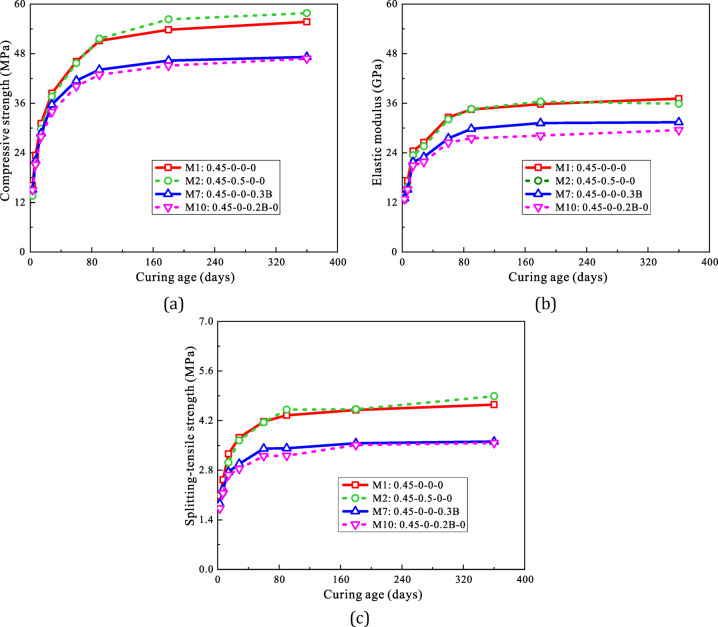
Mechanical property development against with curing age for various LC3-based-RACs: (**a**) Compressive strength, (**b**) Elastic modulus, (**c**) Splitting-tensile strength.

Figure 5a and c show the mean and standard deviation of compressive strength, elastic modulus and splitting tensile strength for four concrete mixtures (M1, M2, M7 and M10) over curing ages ranging from 3 to 360 days. The comparison reveals how metakaolin and limestone powder replacement, as well as the RA replacement rate, affect the temporal development of concrete’s mechanical indices.

In M2, where 50% of the mass was replaced with OPC, the compressive strength at 3, 7, 14, 28, 60, 90, 180 and 360 days was 13.6, 21.4, 29.8, 37.6, 45.7, 51.6, 56.3 and 57.8 MPa, respectively, compared to 16.1, 23.4, 31.1, 38.4, 46.1, 51.1, 53.8 and 55.7 MPa for M1. From 3 to 14 days, M2’s compressive strength was 4.2-15.7% lower than M1, but after 28 days, it began to converge with M1 and was 1.0-4.7% higher at 90 to 360 days. The splitting tensile strength curve showed a similar trend. This was likely due to the slower pozzolanic reaction of metakaolin and limestone powder, which required more time for full hydration.

Over nearly a year, the maximum difference in elastic modulus between M1 and M2 was 8.9%, observed at 7 days. This difference was smaller than the variation in strength, suggesting that concrete deformation was primarily influenced by the aggregates.

Compared to M2, M7 uses 30% coarse RA by volume. Its compressive strength at 3, 7, 14, 28, 60, 90, 180 and 360 days was 15.1, 22.1, 28.7, 35.7, 41.5, 44.1, 46.3 and 47.2 MPa, deviating by 6.4%, 5.7%, 7.7%, 7.0%, 9.9%, 13.7%, 13.9% and 15.2%, respectively, from M2. M7’s elastic modulus at 3-360 days was 13.0, 15.1, 21.8, 23.0, 27.5, 29.8, 31.2 and 31.4 GPa, deviating by 5.7%, 12.3%, 10.6%, 13.1%, 15.6%, 13.5%, 12.8% and 15.4%, respectively. Its splitting tensile strength at 3-360 days was 1.85, 2.26, 2.75, 2.98, 3.41, 3.42, 3.56 and 3.61 MPa, deviating by 11.2%, 10.7%, 15.5%, 19.8%, 18.2%, 21.4%, 20.9% and 22.4%, respectively. Initially, at 3–7 days, the differences between the two mixtures were minimal, with M7 being 5.7-15.5% lower, but the gap widened at 14–360 days, especially in tensile strength. This discrepancy was likely due to the effect of adhered mortar on the RAs. At early ages, the strength of the new mortar did not significantly develop, minimizing the differences. However, as curing progressed, the strength difference between the new and old mortar became more apparent, amplifying the detrimental impact of adhered mortar.

M10, which replaces 20% recycled sand by volume compared to M2, exhibited a similar trend in the relationship between mechanical properties and curing age, as observed with M7 and M2. However, the differences in strength and elastic modulus between M10 and M2 were more pronounced than those between M7 and M2.

### Material property prediction

This section aims to develop predictive models for the mechanical properties of LC3-based RAC. After regression analysis, the following formulas are recommended for predicting the 28-day compressive strength, elastic modulus and splitting tensile strength:

1 $${f_{\text{c}}}=(\frac{{12.23}}{\chi }+11.93)(1 - 7.71 \times {10^{ - 2}}\rho )(1 - 8.15 \times {10^{ - 2}}\eta \frac{{{w_{{\text{FRA}}}}}}{{{w_{{\text{FNA}}}}}})(1 - 7.95 \times {10^{ - 2}}\xi \frac{{{w_{{\text{CRA}}}}}}{{{w_{{\text{CNA}}}}}})$$

2 $${E_{\text{c}}}=(\frac{{7.65}}{\chi }+10.12)(1 - 1.22 \times {10^{ - 2}}\rho )(1 - 0.10\eta \frac{{{w_{{\text{FRA}}}}}}{{{w_{{\text{FNA}}}}}})(1 - 0.12\xi \frac{{{w_{{\text{CRA}}}}}}{{{w_{{\text{CNA}}}}}})$$

3 $${f_{\text{t}}}=(\frac{{0.59}}{\chi }+2.40)(1 - 0.16\rho )(1 - 0.13\eta \frac{{{w_{{\text{FRA}}}}}}{{{w_{{\text{FNA}}}}}})(1 - 0.17\xi \frac{{{w_{{\text{CRA}}}}}}{{{w_{{\text{CNA}}}}}})$$

**Fig. 6:**
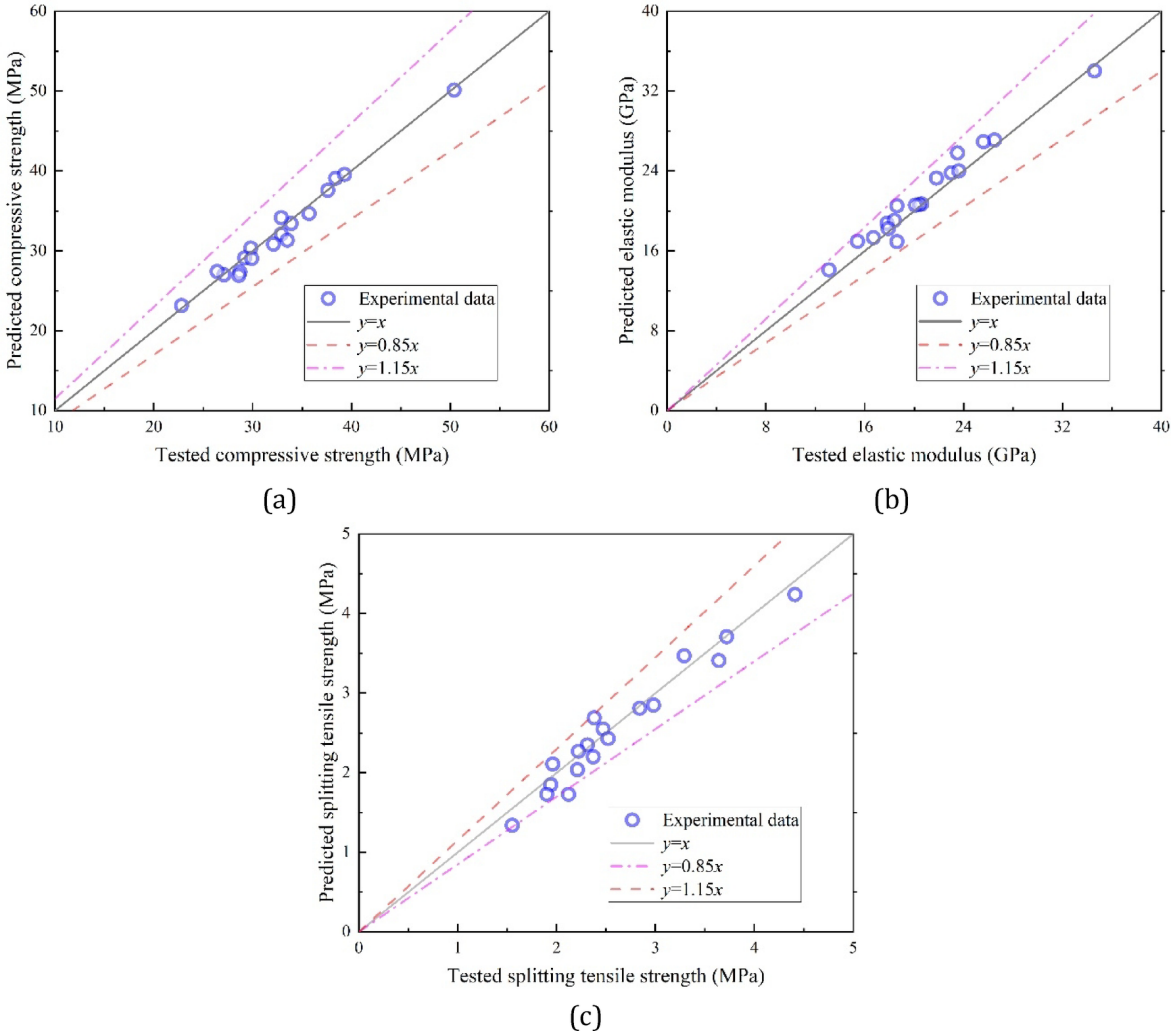
Predicted errors for formulas proposed by this study: (**a**) Compressive strength, (**b**) Elastic modulus, (**c**) Splitting-tensile strength.

In the above equations, *χ*, *ρ*, *η* and *ξ* represent the water-to-binder ratio, the combined replacement ratio of metakaolin and limestone powder, the recycled sand substitution rate and the coarse RA content, respectively, while *w* denotes the aggregate water absorption rate. The deterministic coefficients *R*² are 0.977, 0.965 and 0.950, indicating high prediction accuracy, as shown in Fig. 6. It is important to note that these formulas are currently limited to LC3-based concrete with a cement clinker strength grade of PO52.5.

## Life cycle impact analysis

### Model construction

This section examines the economic and environmental advantages of substituting cement with metakaolin and limestone powder, as well as replacing natural aggregates with RAs. While these substitutions lower concrete costs and carbon emissions, they also result in a reduction in mechanical performance. Consequently, a more in-depth discussion of the “true sustainability” of these materials is warranted. This objective is typically achieved through life cycle assessment, which consists of four key steps: goal and scope Definition, life cycle inventory, life cycle impact assessment and interpretation.

#### Goal and scope definition

The purpose of life cycle assessment is to evaluate the environmental and economic impacts of using LC3 and RAs, while accounting for the influence of various factors on sustainability. This paper introduces two key improvements to the process: (i) Unlike most studies^40–42^, it adopts concrete with equivalent strength per cubic meter as the functional unit, rather than the traditional unit volume of concrete. This is a more realistic approach, as load-bearing capacity is typically the primary requirement in practical applications. (ii) The economic allocation method is used to accurately quantify the energy consumption and carbon emissions associated with recycled component production, ensuring a fair distribution of impacts between waste disposal entities (producers) and recycled material users.

**Fig. 7:**
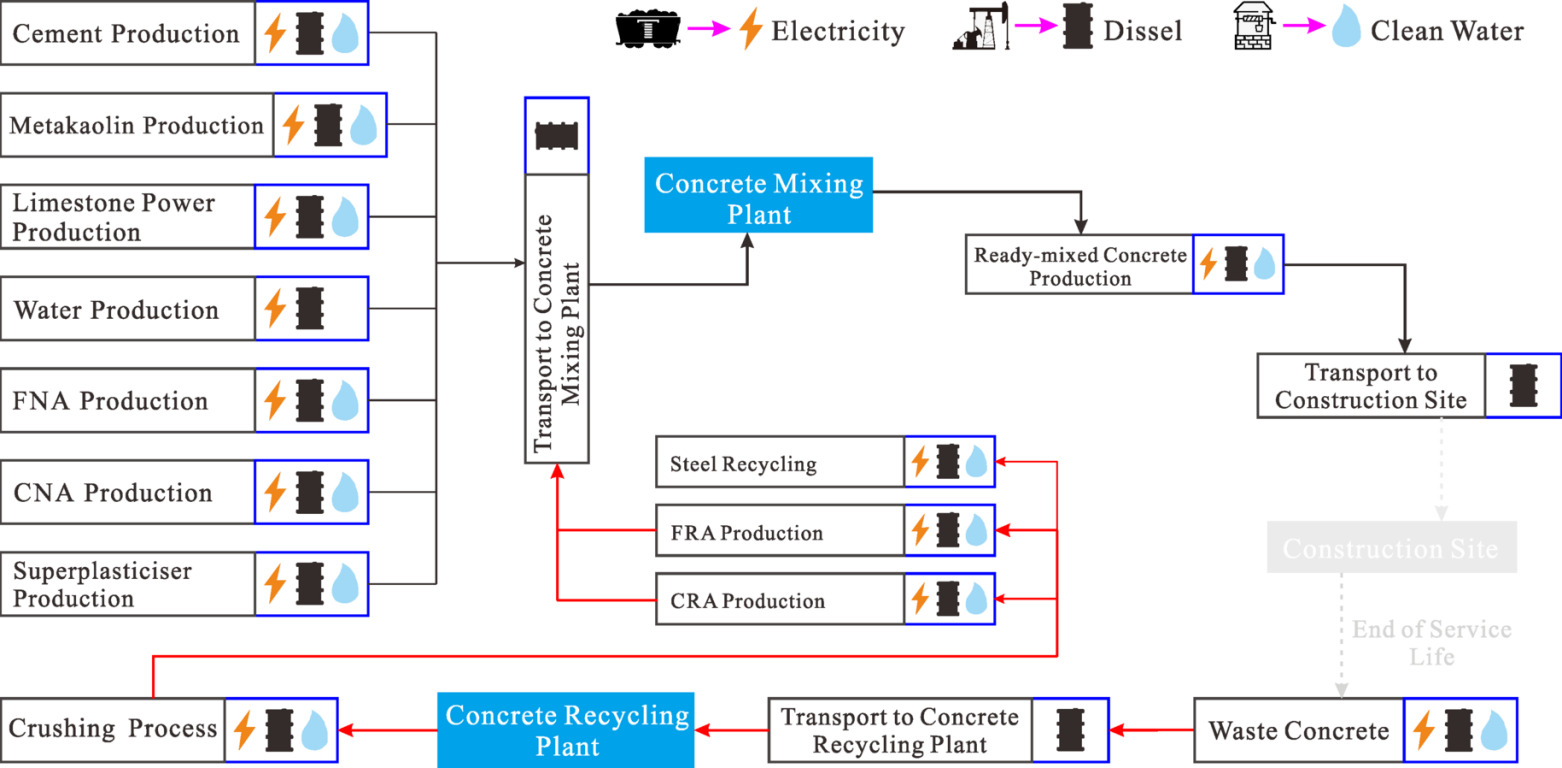
System boundary for the life cycle environmental and cost models.

The solid arrow in Fig. 7 illustrates the system boundary for the lifecycle impact analysis. It is evident that this study adopts a cradle-to-gate approach, encompassing raw material production, secondary processing, transportation, mixing and curing. Due to data limitations, the analysis excludes the operational, reinforcement, repair and demolition stages of the materials once they are formed into components. Based on this approach, the carbon emissions and cost of producing one cubic meter of concrete can be calculated using the following formula^43,44^:

4 $$\begin{gathered} {\text{C}}{{\text{O}}_{{\text{2-eq}}}}={c_{\text{W}}}{m_{\text{W}}}+{c_{\text{C}}}{m_{\text{C}}}+{c_{\text{M}}}{m_{\text{M}}}+{c_{{\text{LP}}}}{m_{{\text{LP}}}}+{c_{{\text{FNA}}}}{m_{{\text{FNA}}}}+{\theta _{{\text{FRA}}}}{c_{{\text{FRA}}}}{m_{{\text{FRA}}}}+ \hfill \\ \;\;\;\;\;\;\;\;\;\;\;\;\;{c_{{\text{CNA}}}}{m_{{\text{CNA}}}}+{\theta _{{\text{CRA}}}}{c_{{\text{CRA}}}}{m_{{\text{CRA}}}}+{c_{{\text{SP}}}}{m_{{\text{SP}}}}+{c_{{\text{mixing}}}}\;\;\;\;\;\;\;\;\;\;\; \hfill \\ \end{gathered}$$

5 $$\begin{gathered} {\text{Cost}}={u_{\text{W}}}{m_{\text{W}}}+{u_{\text{C}}}{m_{\text{C}}}+{u_{\text{M}}}{m_{\text{M}}}+{u_{{\text{LP}}}}{m_{{\text{LP}}}}+{u_{{\text{FNA}}}}{m_{{\text{FNA}}}}\;+\;{u_{{\text{FRA}}}}{m_{{\text{FRA}}}}+ \hfill \\ \;\;\;\;\;\;\;\;\;\;{u_{{\text{CNA}}}}{m_{{\text{CNA}}}}\;+\;{u_{{\text{CRA}}}}{m_{{\text{CRA}}}}+{u_{{\text{SP}}}}{m_{{\text{SP}}}}+{u_{{\text{mixing}}}} \hfill \\ \end{gathered}$$

here CO_2 − eq_ emissions, unit prices and mass quantities of concrete ingredients are denoted by c_*_, u_*_ and m_*_, respectively. The asterisk * refers to water, cement, metakaolin, limestone powder, fine natural aggregates, fine RAs, coarse natural aggregates, coarse RAs and superplasticizer. Specifically, *c*_mixing_ and *u*_mixin_g represent the emissions and cost related to concrete batching. The allocation coefficients for fine and coarse RAs are denoted by *θ*_FRA_ and *θ*_CRA_.

#### Life cycle inventory

Within the framework of the life cycle analysis model, the current study examines a hypothetical scenario for concrete production in the context of a school construction project in Guangzhou, Guangdong. Except for the superplasticizer, sourced from Nanjing, Jiangsu, all raw materials are procured from suppliers in close proximity to the school. The transportation distances for these materials are as follows: cement 37 km, metakaolin 70 km, limestone power 16 km, river sand 65 km, natural coarse aggregate 50 km, recycled sand 60 km and recycled coarse aggregate 60 km, all delivered by freight trucks. In contrast, the superplasticizer is transported via rail freight over a distance of 1300 km.

Tables 3, 4 and 5 present the unit prices and carbon emission values. The material and transportation prices are primarily sourced from references^43,44^ and cost estimation websites^45^, while the carbon emission factors are derived from references^46–49^. It is important to note that, due to data access limitations and the fact that our LCA model is self-compiled, we did not use data from publicly available large LCI databases. This represents a significant limitation of the study.

**Table 3:** Manufacturing cost breakdowns for individual concrete components (CNY/kg).

Year	C	M	LP	W	FNA	FRA	CNA	CRA	SP
2023	0.800	0.400	0.500	0.004	0.143	0.086	0.131	0.092	8.700

(i) C-cement, M-metakaolin, LP-limestone power, W-water, FNA-fine NA, FRA-fine RA, CNA-coarse NA, CRA-coarse RA, SP-superplasticizer; (ii) The batching cost for concrete is 3.6 CNY per cubic meter.

**Table 4:** Per-unit transportation costs for various concrete ingredients (CNY/kg).

Year	C	M	LP	W	FNA	FRA	CNA	CRA	SP
2023	0.013	0.025	0.006	0.000	0.023	0.021	0.018	0.021	0.052

C-cement, M-metakaolin, LP-limestone power, W-water, FNA-fine NA, FRA-fine RA, CNA-coarse NA, CRA-coarse RA, SP-superplasticizer.

**Table 5:** Unit carbon emissions for producing and transporting concrete ingredients (kg/kg).

Ingredient	C	M	LP	W	FNA	FRA	CNA	CRA	SP
Producing	9.12E−1	3.00E−1	1.00E−1	2.56E−4	1.60E−2	3.53E−3	5.27E−2	3.54E−3	1.88E + 0
Conveying	9.47E−3	1.79E−2	4.10E−3	0.00E + 0	1.66E−2	1.54E−2	1.28E−2	1.54E−2	2.32E−2

(i) C-cement, M-metakaolin, LP-limestone power, W-water, FNA-fine NA, FRA-fine RA, CNA-coarse NA, CRA-coarse RA, SP-superplasticizer; (ii) The CO_2 − eq_ emissions for concrete batching are 2.3 kg/m^3^.

**Table 6:** Allocation coefficients for FRA and CRA.

Year	Unit price u (CNY/kg)			Allocation coefficient θ	
	FRA	CRA	Waste disposal	FRA	CRA
2023	0.086	0.092	0.025	29.43%	41.94%

(i) 1.0 kg of waste concrete can produce 0.4 kg of CRA and 0.3 kg of FRA^44^; (ii) *θ*_FRA or CRA_=(*m*×*u*)_FRA or CRA_/[(*m*×*u*)_FRA_ +(*m*×*u*)_CRA_+ (*m*×*u*)_waste disposal_]^44^.

Table 6 presents the calculation method and values for the two allocation coefficients. It is important to note that this study employs an economic value-based approach to allocate carbon emission coefficients. According to the literature^44^, the recycling facility levies a collection fee of 0.025 yuan for every 1.0 kg of discarded concrete, which can be processed into 0.4 kg of CRA and 0.3 kg of FRA. The carbon emissions associated with 1.0 kg of waste concrete are apportioned by allocating 0.4 × 0.092/(1.0 × 0.025 + 0.4 × 0.092 + 0.3 × 0.086) to CRA users and 0.3 × 0.086/(1.0 × 0.025 + 0.4 × 0.092 + 0.3 × 0.086) to FRA users.

### Life cycle impact assessment and interpretation

**Fig. 8:**
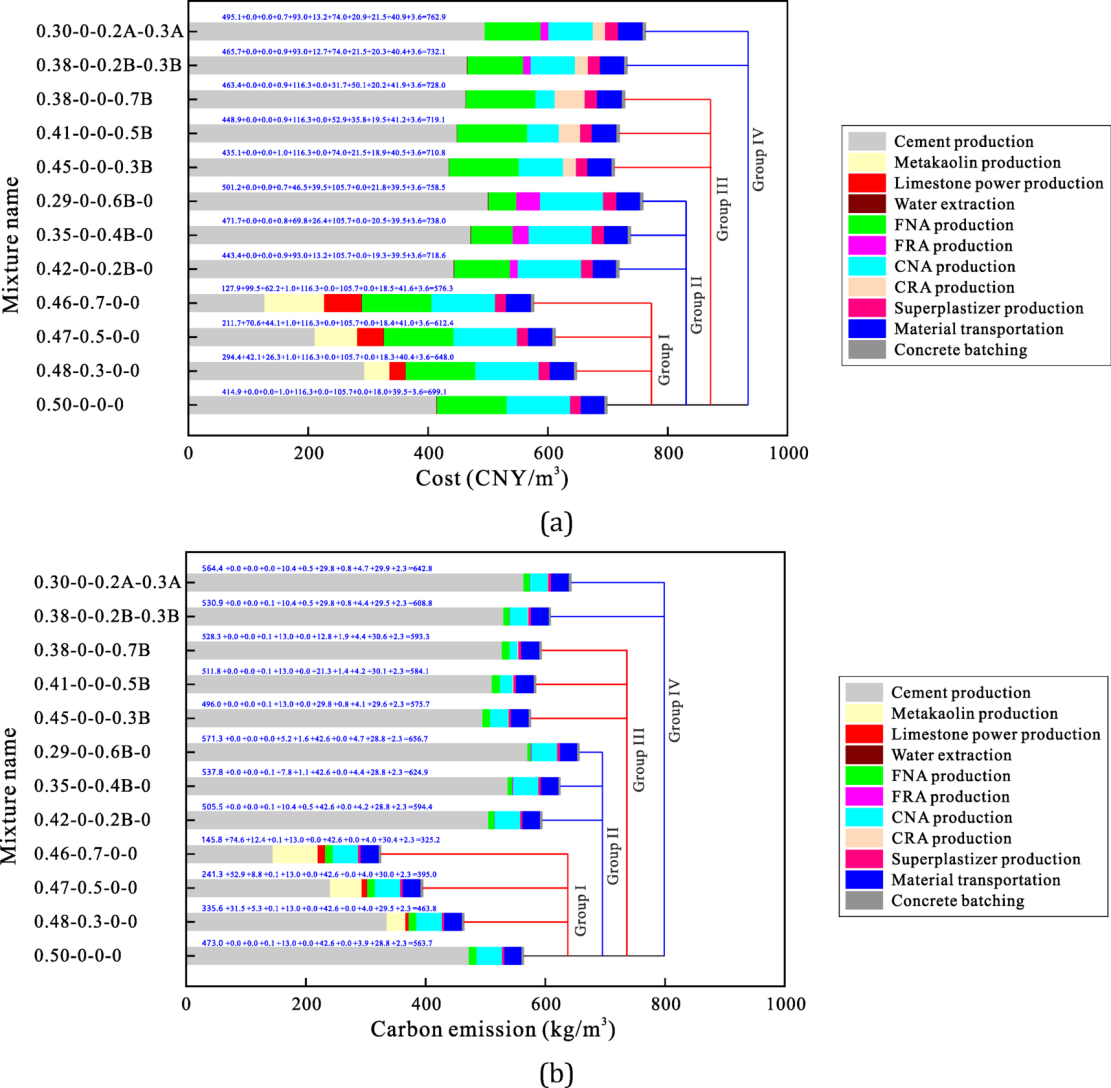
Influences of various internal factors on LC3-based RAC’s production cost and carbon emission: (**a**) Economical cost, (**b**) Carbon emission.

Figures 8a and b show the lifecycle evaluation results, respectively. Both figures feature 4 sets of 12 mix proportions, used to assess the impact of metakaolin and limestone power substitution rates, fine RA content, coarse RA content and RA quality on the carbon emissions and cost indicators of concrete production.

In Group I, the combined substitution rate of metakaolin and limestone powder increases from 0 to 30%, 50% and 70%. Based on earlier findings, to maintain similar 28-day compressive strength, the water-to-binder ratio must be adjusted. Using Eq. (1), the ratio must decrease from 0.50 to 0.48, 0.47 and 0.46 to maintain a compressive strength of approximately 36.4 MPa. The detailed mix ratios and sustainability indicators for the four concrete types reveal the following: (i) For the OPC-based natural aggregate concrete (0.50-0-0-0), the total carbon emissions are 563.7 kg/m^3^, with cement, sand, gravel and superplasticizer accounting for 83.9%, 2.3%, 7.6% and 0.7%, respectively. Emissions from raw material transportation are 5.1% and mixing emissions are 0.4%. The total production cost is 699.1 CNY/m^3^, with cement, water, sand, gravel and superplasticizer contributing 59.4%, 0.1%, 16.6%, 15.1% and 2.6%, respectively. Transportation costs account for 5.6%, and mixing costs 0.5%. Cement production is the most significant factor in determining concrete sustainability, followed by coarse and fine aggregate production. (ii) The use of metakaolin and limestone powder as alternative binders significantly reduces concrete’s production carbon emissions and cost while maintaining mechanical performance. At 30%, 50% and 70% substitution rates, the cost decreases by 7.3%, 12.4% and 17.6%, respectively, while greenhouse gas emissions are reduced by 17.7%, 29.9% and 42.3%. This highlights the strong cost and carbon reduction potential of LC3.

In Group II, the recycled sand content in the concrete increases from 0 to 60%. Similarly, the water-to-binder ratio must be reduced to maintain the same strength. However, due to the significant degradation effect of this material, the water-to-binder ratio reduction is more pronounced, which ultimately impacts the sustainability indicators of the concrete. The simulation results show that the unit cost of natural aggregate concrete is 699.1 CNY/m^3^, while at 20%, 40% and 60% recycled sand substitution, the cost increases to 718.6, 738.0 and 758.5 CNY/m^3^, respectively, rising by 2.8%, 5.6% and 8.4%. Similarly, at these substitution rates, carbon emissions increase by 5.4%, 10.8% and 16.5%. These findings suggest that using recycled sand is not cost-effective or low-carbon, as the increased cement demand outweighs the benefits of the recycled sand.

In Group III, the volume substitution of coarse RAs increases from 0 to 70%. The sustainability indicators of recycled coarse aggregate concrete are nearly identical to those of natural aggregate concrete, with a slight increase of 2-5%. This indicates that coarse RAs can be widely used in load-bearing structures, contributing to the recycling of construction waste.

Finally, Group IV examines the impact of RA particle quality on concrete sustainability. When the parent strength grade of RAs is 21.5 MPa, the unit cost and carbon emissions of RAC increase by 9.1% and 14.0%, respectively, compared to natural aggregate concrete. This underscores the importance of RA quality for concrete sustainability. In practice, low-quality RAs should be avoided for load-bearing concrete, as they are better suited for non-load-bearing applications.

**Fig. 9:**
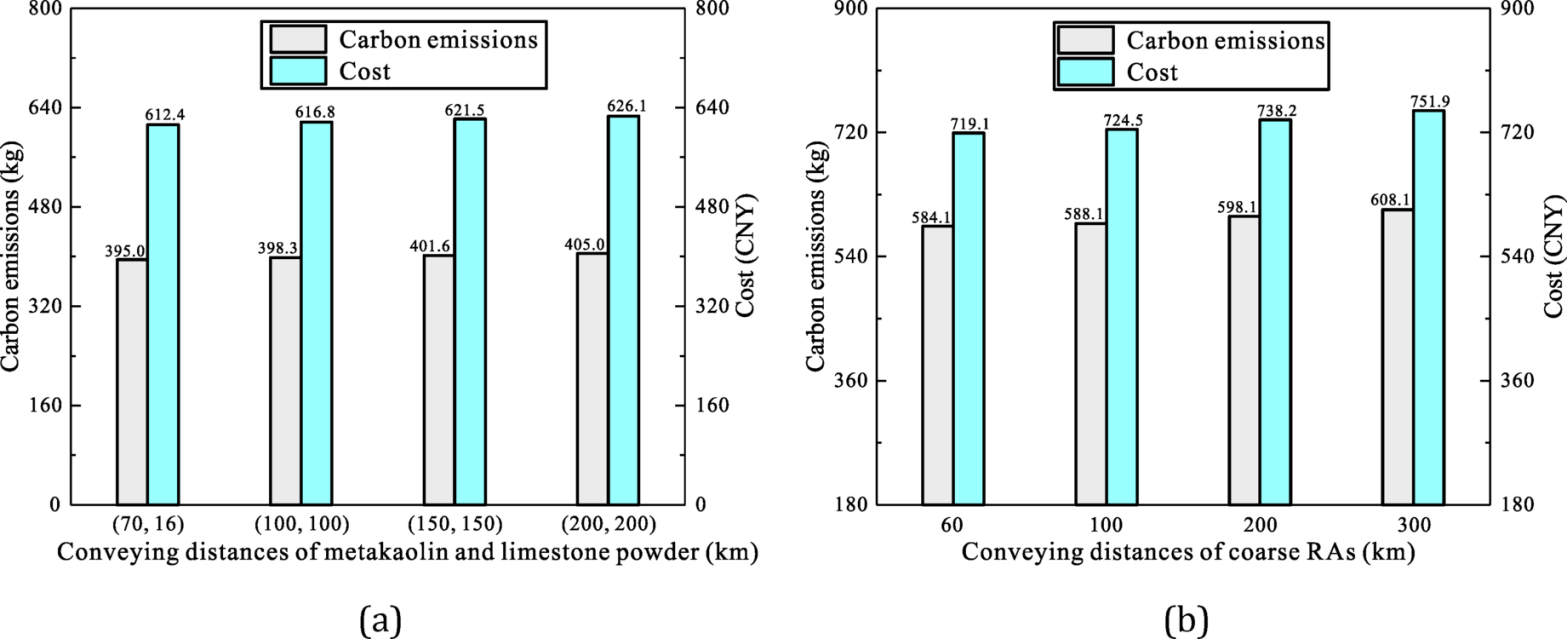
Effect of transportation distance on sustainability of LC3-based-RACs: (**a**) Metakaolin and limestone powder, (**b**) Coarse RAs.

In prior scenarios, transport distances for cement, metakaolin and limestone powder were 37, 70 and 16 km, respectively, exhibiting minor discrepancies. However, supply chain constraints in practice may necessitate sourcing metakaolin and limestone powder from more remote locations. Accordingly, we assessed the impact of transport distance on carbon emissions and costs. With other raw material transport distances fixed, Fig. 9a illustrates variations in these indicators across four scenarios, where metakaolin and limestone powder transport distances extend from 70 to 16 km to 200 km. Even when these distances expand to 5–6 times that of cement, production cost and carbon emissions of concrete 0.47-0.5-0-0 rise only marginally by 2.3% and 2.5%, respectively, while remaining markedly lower than those of OPC-based-concrete 0.50-0-0-0, reinforcing the sustainability advantages of substituting cement with metakaolin and limestone powder. As illustrated in Fig. 8; Table 4, the 0.47-0.5-0-0 mix exhibits nearly 170 kg lower carbon emissions per cubic meter than the 0.50-0-0-0 mix. It contains 176.4 kg of metakaolin and 88.2 kg of limestone, with transport emissions per kilogram per kilometer estimated at 2.6 × 10⁻⁴ kg. Calculations reveal that the allowable transport distance for these materials could extend by at least 2450 km, approximating the maximum internal transport distance within China.

Similarly, Fig. 9b displays the impact of varying transport distances of coarse RA on the production carbon emissions and cost of the RAC mix 0.41-0-0-0.5B. The results reveal a stark shift: when the RA transport distance exceeds that of natural aggregate by 250 km, the carbon emissions and cost per cubic meter of RAC surpass those of natural aggregate concrete by 7.6% and 7.9%, respectively, entirely eroding its economic and environmental benefits. This underscores the necessity for greater scrutiny and strategic planning in the engineering industry.

## Conclusions

LC3 and RAs, as innovative materials in civil engineering, are widely recognized as promising solutions to mitigate the high energy consumption and carbon emissions associated with traditional technologies. However, research on their synergistic application remains limited, hindering their broader adoption in construction. This study systematically investigates the performance of LC3 combined with coarse and fine RAs in concrete, focusing on critical factors such as water-to-binder ratio, metakaolin-limestone powder blend, RA content and particle quality, and their effects on workability, mechanical properties, cost and lifecycle carbon emissions. The findings provide both theoretical insights and practical guidance for material optimization and engineering applications.

Key conclusions are:

- The introduction of metakaolin, limestone power and RAs adversely affects the fresh-state workability of concrete. Specifically, 50% mass replacement of cement with metakaolin and limestone power, 30% volume replacement with coarse RAs, and 20% recycled sand reduce slump by 14.8%, 11.9% and 9.4%, respectively.
- The critical replacement rates for metakaolin-limestone power, coarse RAs and recycled sand are approximately 50%, 30% and 20%, respectively. Exceeding these thresholds results in significant reductions in 28-day strength and elastic modulus.
- At 14 days, LC3-based concrete shows a 4-15% lower absolute strength compared to OPC-based concrete. However, by 28 days, their strengths converge. Concrete with recycled sand or coarse RAs follows a similar early low strength trend, with the performance gap widening as the curing period increases.
- Lifecycle impact analysis reveals that replacing cement with metakaolin-limestone power offers significant sustainability advantages. A 50% mass substitution reduces carbon emissions by 29.9% and lowers unit cost by 12.4%. In contrast, recycling RAs yields limited benefits: 20% recycled sand and 30% coarse RAs increase carbon emissions by 5.4% and 2.8%, respectively, with no significant change in unit cost.
- The RA quality significantly affects sustainability. Lower-quality RAs notably increase carbon emissions and cost in RAC production. Additionally, transportation distance significantly affects the circular benefits of RA use but has a limited impact on the sustainability of metakaolin-limestone powder.

This study has some limitations. Micro-level tests on the microstructure, hydration products and pore structure of LC3-based RAC versus OPC-based natural aggregate concrete were not conducted. Furthermore, durability tests were limited, and the LCA analysis focused solely on the material level. These aspects will be improved in future research by the authors.

## Data Availability

The data that support the findings of this study are available from the corresponding author upon reasonable request.
